# Carotenoid-Based Colours Reflect the Stress Response in the Common Lizard

**DOI:** 10.1371/journal.pone.0005111

**Published:** 2009-04-08

**Authors:** Patrick S. Fitze, Julien Cote, Luis Martin San-Jose, Sandrine Meylan, Caroline Isaksson, Staffan Andersson, Jean-Marc Rossi, Jean Clobert

**Affiliations:** 1 Museo Nacional de Ciencias Naturales (MNCN–CSIC), Madrid, Spain; 2 Instituto Pirenaico de Ecología (IPE–CSIC), Jaca, Huesca, Spain; 3 Laboratoire d'écologie, Université Pierrre et Marie Curie, Paris, France; 4 Department of Environmental Science and Policy, University of California Davis, Davis, California, United States of America; 5 Department of Zoology, Göteborg University, Göteborg, Sweden; 6 Station d'Ecologie Expérimentale du CNRS à Moulis, USR2936, Moulis, Saint-Girons, France; University of Lethbridge, Canada

## Abstract

Under chronic stress, carotenoid-based colouration has often been shown to fade. However, the ecological and physiological mechanisms that govern colouration still remain largely unknown. Colour changes may be directly induced by the stressor (for example through reduced carotenoid intake) or due to the activation of the physiological stress response (PSR, e.g. due to increased blood corticosterone concentrations). Here, we tested whether blood corticosterone concentration affected carotenoid-based colouration, and whether a trade-off between colouration and PSR existed. Using the common lizard (*Lacerta vivipara*), we correlatively and experimentally showed that elevated blood corticosterone levels are associated with increased redness of the lizard's belly. In this study, the effects of corticosterone did not depend on carotenoid ingestion, indicating the absence of a trade-off between colouration and PSR for carotenoids. While carotenoid ingestion increased blood carotenoid concentration, colouration was not modified. This suggests that carotenoid-based colouration of common lizards is not severely limited by dietary carotenoid intake.

Together with earlier studies, these findings suggest that the common lizard's carotenoid-based colouration may be a composite trait, consisting of fixed (e.g. genetic) and environmentally elements, the latter reflecting the lizard's PSR.

## Introduction

**Figure 1:**
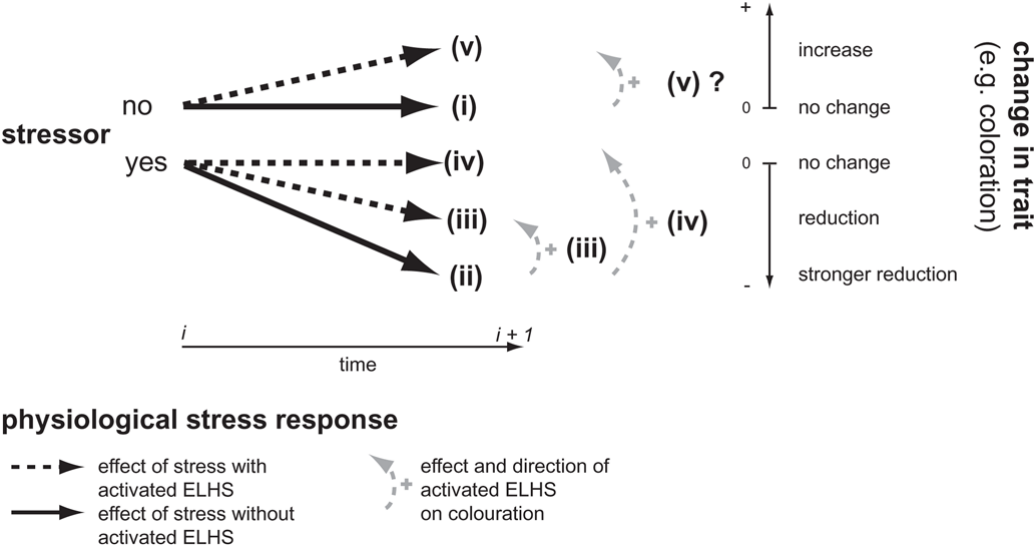
Effects of stress factors and PSR on colouration. The theory predicts that colouration remains unchanged in the absence of stress (i: no difference from time *i* to time *i+1*), while colouration is reduced in the presence of a stress factor (ii: colour reduction from time *i* to time *i+1*). In the presence of a stress factor animals activate the ELHS, thereby decreasing the negative effects of the stress. As a consequence, the colour reduction (ii) between time *i* and time *i+1* will be smaller when the PSR is stronger (iii, iv). If activated ELHS provokes the same effects in the presence and absence of stress factors, as suggested by several studies [30], [42]–[44], activated ELHS may lead to increased colouration (v: colour increase from time *i* to time *i+1*).

Colour signals are usually genetically and/or environmentally determined [1]–[5]. However, while genetically determined elements signal an individual's life history strategy (see [6]–[8] for reptiles and [9] for insects), environmental variance stems from nutritional conditions [1]–[4] and health status [10]–[12]. Environmental determination of many colour signals stems from differences in carotenoid deposition. Carotenoids are widely used colour pigments that cannot be synthesized by animals [13] and thus, they must be obtained through feeding. Carotenoids are relevant to the immune system [2], [14] and they are thought to fulfil antioxidant functions [15], [16] (but see [17]). These multiple functions may create trade-offs in carotenoid-limited animals. For example, during an immune challenge animals may favour using carotenoids for immune function rather than for colouration [2], [18], [19]. Similarly, stress factors may divert the use of carotenoids from colouration by means of allostasis [20], possibly explaining why stressed animals sometimes exhibit reduced colouration [10], [12], [16], [21]–[23] (Figure 1 (ii)). While the trade-off between carotenoid contribution to the immune system and colouration has frequently been investigated, the trade-off between stress and colouration has received much less attention. Indeed, the extent to which such trade-offs occur might depend on the role that colouration plays in a given species: signalling individual quality or individual strategy.

In response to a stress factor, vertebrates usually initiate a physiological stress response (PSR) by secreting glucocorticoids [24], [25]. During a PSR, many vertebrates release corticosterone from the adrenal glands [26], including birds, amphibians, reptiles and some mammals [24], [25], and typically corticosterone concentrations rise within a few minutes in response to acute stress (for example social and agonistic interactions [27]). Corticosterone modifies vertebrate behaviour and physiology (increasing oxygen intake, decreasing pain perception, and enhancing sensory function and memory [25], [28], [29]). It also acts on the metabolic pathways to replenish the energy reserves used during stress [25], [30], for example when escaping a predator. In addition, it increases locomotor performance [30], [31], which may facilitate the possibility of finding new food sources [31], suggesting that at least during acute PSR corticosterone adaptively influences vertebrate behaviour [32], [33]. During acute stress, increased blood corticosterone levels are typically associated with an increase in oxidative stress [34], suggesting that carotenoids may be used as antioxidants against oxidative stress and that corticosterone may reduce carotenoid-based colouration (through its effect on oxidative stress: Figure 1 (ii)).

In contrast to acute stress, chronic stress has very different implications since it may change an animal's life history strategy from long-term to short-term investment [35]. Such a change may have potentially important consequences that include reproductive suppression [36], reduced immunocompetence [37], [38], decreased insulin production, neural degeneration [39] and reduced colouration (Figure 1 (ii)). However, chronic stress produced by unpredictable adverse longer-term conditions (perturbation factors) such as drought, changes in social status, increased predator number, decreased food resources and diseases, is also responsible for animals entering an emergency life history stage (ELHS [32]), whereby animals may try to reduce the negative effects of a stress. The ELHS theory may explain why in some species increased blood corticosterone levels due to chronic stress do not necessarily increase oxidative stress [40], and why a blood corticosterone increase may even reduce oxidative stress during chronic stress [41]. Hence, chronic stress is likely to affect colouration differently than acute stress. These findings are in line with studies showing that chronic corticosterone elevation positively affects survival [30], [42]–[44]. Indeed, they suggest that the negative effects of glucocorticosteroids may be compensated for (Figure 1 (iii)), or even avoided [32] (Figure 1 (iv)) if an animal is able to compensate the perturbing effects of a stress factor by activating the ELHS. These studies also suggest that successful compensation may depend on the presence of abundant food. Indeed, most studies providing evidence of the beneficial effects of chronic corticosterone exposure were carried out on well-fed individuals, potentially hiding the negative effects of elevated corticosterone concentrations [45].

Disentangling the effects of a stress factor and the effects of the ELHS is not a simple task, since blood corticosterone levels are normally not increased and the ELHS is not activated in the absence of stress. Consequently, experimental designs are necessary that exclude the potentially negative effects of the stress factor and that activate the ELHS. For example, if parasite-induced stress provokes negative effects (Figure 1 (iv)) the ELHS or the stress factor itself may be responsible for the negative outcome. In other words, if parasite abundance causes a negative effect and if the ELHS is the adaptive response to the stress, positive effects of ELHS may exist in the presence of abundant food (outcome (v) in Figure 1), and negative effects in the absence of abundant food (outcome (ii)). However, if the ELHS is the cause of the negative effects, a negative change similar to outcome (ii) might occur, while outcome (i) would be expected if the ELHS were neutral.

Here, we investigated the effects of increased PSR on colouration in the presence of abundant food and thus, on the signal content of carotenoid-based colouration in the common lizard (*Lacerta vivipara*). First, we investigated the association between natural baseline corticosterone concentrations and colouration. Subsequently, we investigated the effects of corticosterone during chronic stress and its effects on carotenoid-based colouration on animals fed *ad libitum*. We predicted a negative colour change in corticosterone-treated lizards if increased blood corticosterone levels were to invariably lead to colour reduction (similar to Figure 1 (ii)). If chronic corticosterone elevation activates the ELHS and the ELHS can compensate in the presence of abundant food, as suggested by some experimental studies [30], [42]–[45] (but see [46]), we predicted no colour change (Figure 1 (i)) or even a positive colour change in corticosterone treated animals (Figure (v)) due to the lack of an external stress.

Finally, we investigated the pigments responsible for the common lizard's colouration and tested whether common lizards are carotenoid limited. We also assessed whether limited carotenoid affects colouration by feeding half of the individuals in each corticosterone group with additional carotenoids. We predicted that lizards fed with carotenoids will have higher blood carotenoid levels and increased colouration when compared to the carotenoid limited group. This approach also allowed us to test whether a trade-off existed for carotenoids between colouration and the PSR (i.e. if the PSR alters the carotenoid allocation through allostasis [25]). If increased blood corticosterone levels were to have negative effects on colouration through their effect on carotenoid availability, we would predict that carotenoid limited lizards should show a colour reduction while carotenoid unrestricted lizards (the carotenoid supplemented animals) would show no or a smaller reduction in colour.

## Materials and Methods

### Species description

The common lizard (*Lacerta vivipara*) is a small ovoviviparous Lacertidae that inhabits peat bogs and moist heathland. The ventral colouration of males ranges from yellow to red with dark spots. In females, the ventral colouration ranges from cream to orange with only a few dark spots [47], [48]. After birth, juveniles are melanic and they start developing their yellow-orange colouration during their first year of life. The fully developed colouration usually appears after the first or second hibernation [48]. Adult colouration is determined both genetically and environmentally [23], [48]. During long-lasting stressful situations (e.g. increased density), the colouration of the common lizard fades and becomes less red [22], [23]). The function of ventral colouration has rarely been investigated in this species, although it has been shown experimentally that it plays an important role in courtship. Indeed, male common lizards that were painted with adult female colour patterns were courted like adult females [47], [49]. More recent experiments show that ventral colouration plays an important role during sexual selection, given that females choose their mating partners based on this colouration (Fitze et al., submitted). The common lizard's yellow-orange ventral colouration is thought to stem from carotenoids [50]. To confirm the basis of these colours and to investigate whether other pigments might also participate in producing the yellow-orange colouration (for example pteridines), we first analysed the pigments responsible for the ventral colouration. Thereafter, we investigated whether ventral colouration might be correlated with certain individual characteristics as seen in many other species.

### Determination of colour pigments

#### Sample collection

To analyze which pigments were responsible for the yellow–orange belly colouration of the common lizard, we analyzed the skin of nine adult male common lizards originating from an experimental population at the Ecological Research Station of Foljuif (Seine-et-Marne, 48°17′N, 2°41′E). After capture, the males were decapitated and the ventral skin was detached from the muscles. Thereafter, the skin was weighed and individually stored in 1 ml acetone at −80°C until it was analysed.

#### Carotenoid extraction and HPLC analysis

Carotenoids were extracted from the skin, the acetone solution in which they were stored, and from plasma samples. The skin was washed in hexane, dried, weighed and homogenized in methanol in a Retsch MM 2000 micronizer at 27 Hz for 15 minutes (Hann, Germany) with ZrO containers. The residue was filtered (GHP Acrodisc 13 mm) and the methanol was evaporated (ThermoSavant SPD111V, New York, USA). The carotenoid residue was finally dissolved in 10 µl tetrahydrofuran (THF) and 90 µl of the mobile phase (70∶30 acetonitrile∶methanol), and it was immediately analyzed by High Performance Liquid Chromatography (HPLC, see below).

The sample (60–80 µl) was injected with an isocratic mobile phase onto a RP-18 column (YMC Europe GmbH, Schermbeck, Germany) fitted to a ThermoFinnigan (San Jose, USA) HPLC system with a PS4000 ternary pump, an AS3000 auto sampler, and an UV6000 diode-array UV/VIS detector. Chromatograms were obtained and analyzed with ChromQuest 4.0 software (ThermoFinnigan, San Jose, USA). The carotenoids were identified and quantified using lutein and zeaxanthin standards (Roche Vitamims Inc., Basel, Switzerland). Other carotenoids, such as astaxanthin, were compared (or matched) to previously run standards stored in the Chromquest library on the basis of the absorbance peak, lambda max and retention time. Carotenoids were only confirmed when they could be matched to the standards with a confidence limit of 99.9%. All concentrations were calculated as µg/g dry skin.

### Measurement of baseline corticosterone levels in semi-natural and natural populations

#### Sample collection

We used blood samples from 256 adult lizards (68 females and 188 males) from natural (*N* = 69) and semi-natural populations (*N* = 187) to investigate the baseline corticosterone levels and their association with colouration. Individuals from natural populations were captured in May and June (2003 and 2004) from four different lizard populations on the Mont-Lozère in the Cévennes (1500 m asl., Massif Central, Southeastern France, 44°00′N, 3°45′E), while individuals from semi-natural populations at Foljuif were captured in May 2003. Apart from the standard measurements made on all lizards, spectroradiometric measurements were obtained from the lizards from the semi-natural populations. Blood samples were taken on all the lizards immediately after capture and they were conserved at −20° until they were analysed.

#### Corticosterone measurement

Blood corticosterone concentrations were determined using a single enzyme-immunoassay procedure (IDS Inc, USA octeia corticosterone kit, ref AC14F-1, lot 55642). In brief, this kit is a competitive enzyme-immunoassay utilizing a polyclonal antiserum against corticosterone coated onto the inner surface of polystyrene microtitre wells. Calibrators, controls and samples were incubated overnight at 28°C with peroxidase-labelled corticosterone in the antibody-coated wells. The wells were then washed and a colour reaction was developed using the tetramethylbenzidine chromogen. The absorbance of the reaction mixtures were read in a microplate reader, whereby the colour intensity developed was inversely proportional to the concentration of corticosterone in the samples. The blood corticosterone levels found were highly repeatable (correlation across individual repeats, n = 4; *F*_1,2_ = 26.71, *P* = 0.036, *r* = 0.93).

### Effects of blood corticosterone levels and carotenoid availability on belly colouration

#### Pre-experimental procedures

Early in July 2002, we captured 88 adult males and 88 adult females from the four natural populations mentioned above. Lizards were moved to the laboratory at the Ecological Research Station at Foljuif (Seine-et-Marne, 48°17′N, 2°41′E) where they were individually housed in terrariums (terrarium size: 25×15×15 cm) under standard conditions (heat, light, water and food). Terrariums were heated on one side with a bulb (25W) from 0900 to 1200 h and from 14:00 to 17:00 (for details see [51]). The lizards were randomly distributed with respect to body size and population of origin within the laboratory (all *P*>0.6). We measured the snout-vent length (SVL) and the tail length of each lizard to the nearest 1 mm. SVL is a determinant of mating success [52], [53] and intra-sexual interactions [52], and the tail length reflects energy storing capacity since lizards use the tail for fat storage [54]. Body mass was measured to the nearest 0.002 g, and the colouration was measured as indicated below.

#### Experimental procedures

The experiment started on August 2^nd^, 2002 (hereafter referred to as day one) when the lizards were randomly assigned to the different treatments. The body mass of each lizard was measured on the second and the 25^th^ day of the experiment.

#### Corticosterone application

We randomly assigned half of the lizards from each population and sex to a corticosterone group on day one (12 lizards per sex in three of the four populations and 8 lizards per sex in the fourth population), and the remaining lizards were assigned to a control group. The corticosterone treatment consisted of a daily application of 4.5 µl of sesame oil mixed with corticosterone (3 µg of corticosterone/µl oil [55]). Control lizards were treated with 4.5 µl of sesame oil alone [30], [56]. The treatment was applied each evening to the lizards back to simulate chronic stress, commencing on the second day and ending on day 23 [56].

#### Carotenoid supplementation

On day one, half of the corticosterone treated and control treated lizards were assigned to a carotenoid supplementation group while the other half was assigned to a control group using a crossed two-factorial design. For each population and sex, the same number of lizards was attributed to one of the four treatment combinations. There were no significant differences at the start of the experiment in SVL, neither between carotenoid-treatment groups (F_1,172_ = 0.053, P = 0.819) nor between corticosterone-treatment groups (F_1,172_ = 0.014, P = 0.905), and the interaction between both was not significant (F_1,171_ = 0.040, P = 0.841). Similarly, there were no differences in the initial body condition among treatment groups (corticosterone- treatment: F_1,171_ = 0.010, P = 0.919; carotenoid-treatment: F_1,171_<0.001, P = 0.992; interaction: F_1,170_ = 0.304, P = 0.582). Lizards of the carotenoid supplementation group were administered the carotenoids lutein, zeaxanthin and ß–carotene, the carotenoids that are naturally ingested when eating lepidopteran larvae [57]. The carotenoid ratio (lutein: 77.4%, zeaxanthin: 6.1%, and ß–carotene: 16.5% of total carotenoid content) with which lizards were fed was similar to that found in lepidopteran larvae [57]. We diluted 818 mg lutein/zeaxanthin beadlets, containing 5.58% lutein and 0.44% zeaxanthin, and 130 mg ß–carotene beadlets (containing 7.5% ß–carotene) in 100 ml H_2_O (total 0.5899 mg carotenoids/ml H_2_O). Then, 0.03 ml of this solution (0.018 mg carotenoids) was injected with a sterile syringe into a moth larva (containing on average 1.6 µg/g carotenoids). The lizards were fed a single moth larva every five days and thus, carotenoid supplemented lizards ate between 0.018 mg and 0.106 mg carotenoids. Common lizards can eat more than nine moth larvae (average weight 200 mg) in 24 days and lepidopteran larvae, which are part of their natural diet, contain an average of 0.0033 mg carotenoids/g larvae [57]. Thus, our carotenoid treatment corresponds to high doses ingested under natural conditions, which may compensate for the effects of the corticosterone treatment. Prior to supplementation, we injected the control moth larvae with 0.03 ml of a solution consisting of 948 mg control beadlets dissolved in 100 ml H_2_O. Control beadlets contained of the same ingredients as the carotenoid beadlets except for the absence of carotenoids. All lizards were fed a single moth larva every five days, starting on the third day of the experiment and ending on the 23^rd^ day of the experiment. Only larvae of similar body mass were used (254 mg±12.64 SE). Live larvae were presented to the lizards between 11:30 a.m. and 12:30 p.m and the lizards either accepted the food immediately (attacked and ate the larvae) or they refused it. In the latter case, we left the larva in the terrarium and checked in the evening whether the larva had been eaten. If not, it was removed from the terrarium. This procedure avoided confounding a lizard's appetite with a refusal due to human induced stress and it made sure that the exact number of larvae eaten during the entire experiment was determined.

### Effects of corticosterone and carotenoid treatment on blood corticosterone and blood carotenoid concentrations

#### Effect of corticosterone application on blood corticosterone concentrations

Given that it was impossible to take blood samples without the risk of affecting the experimental outcome, we repeated the corticosterone treatment in 2006 using 36 males (captured from the same populations). This experiment enabled us to test the effects of the corticosterone treatment on blood corticosterone levels. Blood samples were taken at the end of the treatment and conserved at −20° until they were analysed.

#### Effect of carotenoid ingestion on blood carotenoid concentration

To verify, whether the carotenoids ingested increased the blood carotenoid concentration, we conducted a carotenoid feeding experiment in 2007. Prior to the experiment we captured 14 common lizard males and transferred them to the lab. Seven randomly chosen male lizards were fed with carotenoids and another seven males were not given the cartenoid supplement according to the aforementioned protocol. After 20 days we took a blood sample (10 µl) that was centrifuged and stored at −80° until the blood carotenoids were analysed.

### Colour measurement

Before instigating the experimental procedures and after they were terminated, the lizard's belly colouration was measured over the 300 nm to 700 nm visual spectrum at a 45° angle using a miniature spectroradiometer (USB2000, Ocean Optics Inc., Dunedin, FL, USA) and a Xenon light source (PX-2 and R400-7-UV/VIS, Ocean Optics Inc). Reflectance was measured in relation to a diffuse white standard (WS-1, Ocean Optics Inc.) uniformly reflecting 98–100% over the spectral range. For each lizard, we took a colour measurement on the breast, in the middle of the belly and on the anal plate, each corresponding to the average colouration of a surface of approximately 1 mm^2^, avoiding black spots. Average colouration per lizard was used for the analysis.

**Figure 2:**
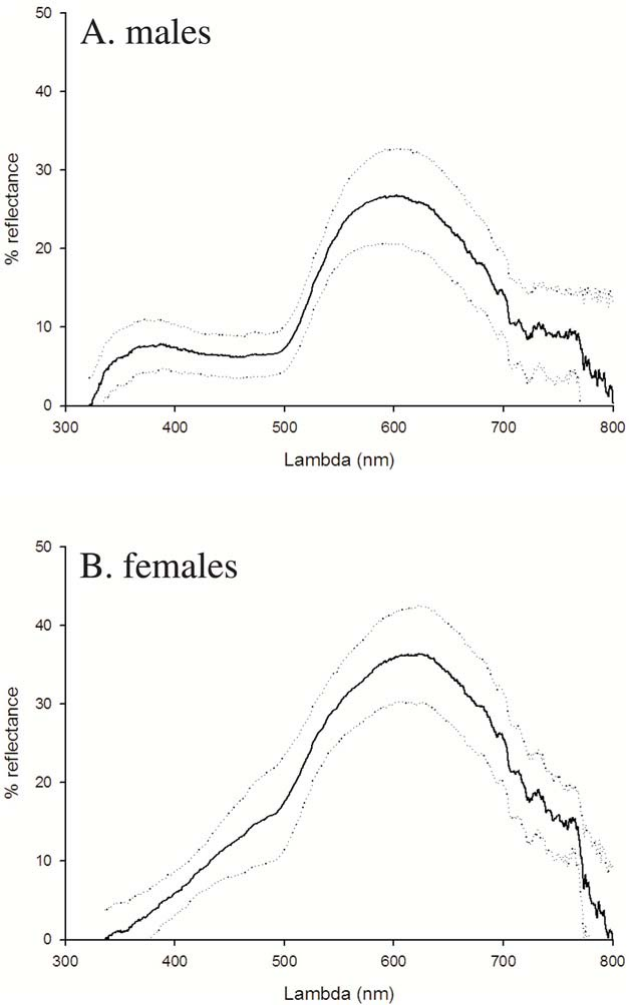
a–b. Average reflectance spectra of ventral colouration in male (a) and female (b) common lizards. The mean and SD per sex of the reflectance spectra measured at the beginning of the experiment are given.

Since saturated carotenoid (and melanin) pigmentation removes most of the reflectance in the ultraviolet wavelengths [5] (Figure 2), we restricted the analyses to the human visible spectrum (400 nm–700 nm). Using Endler's [58] segment classification method, we derived objective estimates of hue (0–360°: 0° = red; 60° = yellow), chroma (0–100%), and brightness (0–100%), and we used the mean colouration of the three body parts measured in the analyses. The colour measurements taken three times from 218 lizards originating from the same populations were repeatable [53] (hue: *F*_216,434_ = 6.99, *P*<0.0001, *r* = 0.66; chroma: *F*_217,437_ = 7.01, *P*<0.0001, *r* = 0.67; brightness: *F*_217,437_ = 10.5, *P*<0.0001, *r* = 0.76; [59]).

### Statistics

To analyze whether specific traits were associated with the colouration we applied mixed model ANCOVAS. In these statistical models we included: population as a random factor; sex as a fixed factor; the covariates SVL, tail length and body mass; and the interactions with sex. The effects of carotenoid and corticosterone treatment on the change in the lizard's ventral colour (after minus before the experiment) were analyzed using mixed model ANCOVAS with sex, carotenoid and corticosterone treatment as fixed effects, population as a random effect, and food consumption and body mass change (final body mass – initial body mass) as covariates. For the analysis of the treatment effects on the body mass change, we applied ANOVA and for the effects on appetite, we applied a GLIMMIX with a Poisson error and a log link using SAS v8.2.0. [60]. For the analyses of body condition, we simultaneously introduced body mass and SVL into the model. Therefore, body condition is a measure of the size-corrected body mass. For all the analyses, the full model included all the parameters and their interactions, and the final model was determined using backward elimination. The assumptions of the models applied were tested and they were met in all the analyses presented (e.g. for parametric tests: equal variances among factor levels and normality of the residuals). All measurements were made blindly with respect to the treatments and the significance level was set at *P*_two-tailed_≤0.05 for all tests. One control treated female died during the experiment and was therefore excluded from the analyses.

## Results

### Pigments responsible for the yellow-orange belly colouration

The yellow-orange colour disappeared from the skin within one hour when it was immersed in acetone and it adopted a bluish colour. This shows that the yellow-orange skin colour of common lizards stem from carotenoids and not from melanins or pteridins, neither of which are soluble in acetone [61]. In total, the skin contained 54.09 µg±4.67 SE carotenoids per gram tissue (5.144 µg±0.467 SE carotenoids per gram fresh weight), including lutein, zeaxanthin, astaxanthin, and canthaxanthin. Based on the dry weight of the skins (0.031 g±0.002 SE), we estimated that the yellow-orange belly colour derives from an absolute amount of 1.641 µg±0.149 SE of carotenoids. The skin's carotenoid concentration was negatively correlated with the lizard's hue (*t*_1,7_ = −2.50, *P* = 0.041, *r* = −0.687), which was measured prior to decapitation, and the skin's carotenoid concentration explained 47.2% of the variance in hue. There was no significant correlation between the lizard's chroma (*t*_1,7_ = 1.45, *P* = 0.191, *r* = 0.480) or the lizard's brightness (*F*_1,7_ = 1.58, *P* = 0.159, *r* = 0.512) and the lizard's skin carotenoid concentration. However, the sample size was small (*N* = 9) and thus, care must be taken in interpreting these results.

### Belly colouration in relation to sex and phenotypic traits

Males (*N* = 88) had higher chroma (male: 0.325±0.007 SE, female: 0.268±0.008 SE) and lower brightness (male: 0.141±0.003SE, female: 0.218±0.003 SE) values than females (*N* = 87, Table 1), and there were no sex differences in the hue. There was a negative correlation between body mass (estimate: −3.905±1.586 SE) and hue, indicating that individuals with greater body mass were redder in colour (Table 1). Tail length, body size and the population were not correlated with hue (Table 1). When co-linearity was assessed by including and excluding the different covariates in distinct orders, the final models were the same as those presented in Table 1. The quadratic terms of body size, tail length and body mass, and their interactions were not significant (*P*>0.1).

**Table 1:** Effects of sex, body size and body mass on colouration of 175 common lizards caught in natural populations.

Variable	hue		chroma		brightness	
	test statistic	*P*	test statistic	*P*	test statistic	*P*
sex	*F*_1,170_ = 0.458	0.499	***F*** **_1,173_** = **29.422**	**<0.001**	***F*** **_1,173_** = **327.894**	**<0.001**
SVL	*F*_1,171_ = 0.802	0.372	*F*_1,169_ = 0.746	0.389	*F*_1,172_ = 1.595	0.208
tail length	*F*_1,172_ = 2.872	0.092	*F*_1,168_ = 0.256	0.614	*F*_1,171_ = 0.869	0.353
body mass	***F*** **_1,173_** = **6.066**	**0.015**	*F*_1,167_ = 0.048	0.828	*F*_1,170_ = 0.024	0.878
population	*F*_3,167_<0.001	1.000	*F*_3,170_ = 1.564	0.200	*F*_3,167_<0.001	1.000

The results from an ANCOVA analysis are shown with: hue, chroma and brightness as dependent variables; sex as a fixed factor; population as a random factor; and SVL, tail length and body mass as covariates. The final model was determined using backward elimination and is shown in bold. Test statistics of backward eliminated variables are given before backward elimination.

### Natural blood corticosterone levels

The mean basal blood corticosterone levels were 49.93±39.14 ng/ml (range: 1 to 185 ng/ml). Body condition was positively correlated with the blood corticosterone levels (*F*_1, 253_ = 15.902, *P*≪0.001; 6.4% of the variance explained; estimate: 2.690±0.603 SE [log(corticosterone level)^2^ transformed data]), but this was not the case for body size (*F*_1, 251_ = 0.875, *P* = 0.351). There were no significant differences in blood corticosterone levels between sexes (*F*_1, 252_ = 0.256, *P* = 0.613). Lizards from experimental populations had lower blood corticosterone levels than lizards from natural populations (*F*_1,253_ = 52.969, *P*≪0.001; 17,04% of variance explained; estimate [experimental populations]: −2.898±0.398 SE).

**Figure 3:**
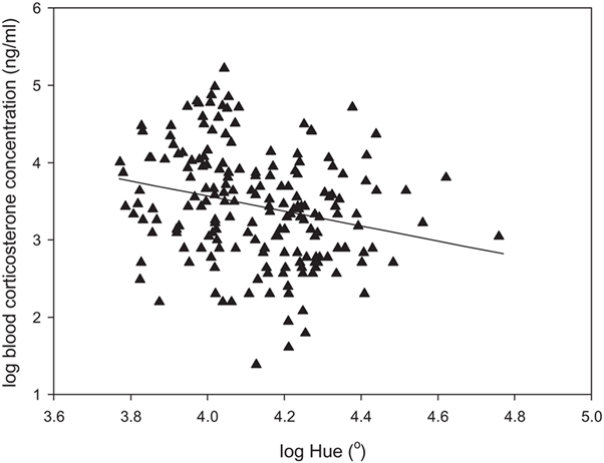
Correlation between basal blood corticosterone levels and the hue of the ventral colouration. The data were loglog transformed to meet the assumptions of the statistical analyses.

Hue was negatively correlated with the blood corticosterone levels (loglog transformed data: *F*_1, 185_ = 11.357, *P*<0.001, estimate: −0.971±0.288 SE; Figure 3), while chroma was not correlated (*F*_1, 185_ = 0.0003, *P* = 0.99, estimate: 0.000±0.000 SE). In addition, there was a trend for brightness to be positively correlated with blood corticosterone levels (*F*_1, 185_ = 3.59, *P* = 0.06, estimate: 5.800±3.061 SE).

### Effects of corticosterone and carotenoids on blood content and belly colour

Blood corticosterone concentrations in corticosterone-treated males were significantly higher (138.23 ng.ml^−1^±16.86 SE) than those of placebo-treated males (86.73 ng.ml^−1^±15.93 SE: *F*_1, 34_ = 5.02, *P* = 0.03, 25.75±11.50 SE). Hence, the blood corticosterone concentrations following corticosterone administration were within the naturally occurring range (see paragraph on natural blood corticosterone levels).

**Figure 4:**
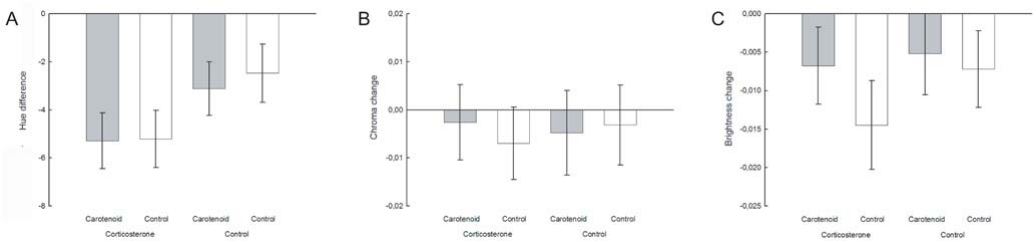
a–c. Change of the lizard's belly colouration in (a) hue, (b) chroma and (c) brightness in relation to carotenoid and corticosterone treatment. Raw means (±SE) of the colour change (colour after treatment – colour before treatment) are shown and Table 1 shows the statistical analysis.

During the experiment, the mean hue of the lizard's ventral colouration decreased from 71.6±0.8 SE to 67.6±0.7 SE, that is towards a redder colour (Figure 4A, *P*≤0.046) and corticosterone treated lizards became significantly redder than control lizards (Table 2, Figure 4A). The effect of corticosterone on hue was significant in all models of backward elimination, but it did not affect chroma or brightness (Table 2, Figures 4B and 4C). There were no significant interactions between corticosterone treatment and any of the other parameters (all *P*>0.1). Furthermore, sex, population, food consumption and change in body mass did not significantly affect the colour change. Carotenoid supplementation led to an increase in blood carotenoid concentration (lutein: *F*_1,12_ = 17.567, *P* = 0.001, estimate = 7.526±1.796 µg/ml, R^2^ = 0.59; zeaxanthin: *F*_1,12_ = 12.443, *P* = 0.004, estimate = 0.47±0.133, R^2^ = 0.51), but it did not affect ventral colouration (Table 2, Figure 4A–C). Moreover, there were no significant interactions between carotenoid treatment and the other parameters (all *P*>0.1). The changes in both chroma and brightness of all treatment groups were no different from zero (Figure 4B, C, all *P*≥0.16). Over the course of the experiment, lizards ate an average of 3.989 larvae±0.0 SE and thus the carotenoid fed lizards consumed an average of 0.071 mg±0.002 SE carotenoids. The average amount of carotenoids ingested corresponded to 43 times the amount of carotenoids responsible for the ventral colouration, and around 4 times the amount of carotenoids present in the skin, intestine and liver [50], [62].

**Table 2:** Effects of carotenoid supplementation and corticosterone treatment on colour change in hue, chroma and brightness.

Variable	hue		chroma		brightness	
	test statistic	*P*	test statistic	*P*	test statistic	*P*
corticosterone	***F*** **_1,168_** = **8.549**	**0.004**	*F*_1,161_ = 0.030	0.863	*F*_1,161_<0.01	0.989
carotenoid	*F*_1,164_<0.01	0.981	*F*_1,162_ = 0.225	0.636	*F*_1,168_ = 1.588	0.209
sex	*F*_1,167_ = 1.210	0.273	*F*_1,166_ = 0.600	0.440	*F*_1,165_ = 0.200	0.655
number larvae eaten	*F*_1,166_ = 2.430	0.121	*F*_1,167_ = 2.986	0.086	*F*_1,166_ = 0.183	0.669
body mass change	*F*_1,165_ = 0.157	0.693	*F*_1,168_ = 0.779	0.379	*F*_1,167_ = 1.735	0.190
population	*F*_3,161_<0.01	0.981	*F*_3,163_ = 0.901	0.442	*F*_3,162_ = 0.074	0.974

The results shown are of an ANCOVA analysis with: hue, chroma and brightness as dependent variables; sex as a fixed factor; population as a random factor; and the number of larvae eaten and the body mass change as covariates. The final model was determined using backward elimination and is shown in bold. Test statistics of backward eliminated variables are given before backward elimination.

### Effects of corticosterone and carotenoids on body mass and appetite

Corticosterone-treated lizards had enhanced appetite (corticosterone treated: 3.44±0.04, control: 3.12±0.05 larvae eaten, *F*_1,172_ = 5.95, *P* = 0.016), and there were no differences in appetite between carotenoid-fed and control-fed lizards (*F*_1,157_ = 0.06, *P* = 0.805; estimate: −0.010±0.040 SE). The interaction between carotenoid and corticosterone treatment was not significant (*F*_1,137_<0.01, *P* = 0.958) and likewise, population and all other interactions did not significantly affect appetite (*P*>0.2). Females ate more than males (*F*_1,172_ = 33.28, *P*<0.001, estimate: 0.235±0.041 SE) and as such, the females increased their body mass while the body mass of the males remained almost constant (*F*_1,173_ = 27.66, *P*<0.001, estimate: 0.055±0.010 SE). The change in body mass did not differ between corticosterone and control administered (*F*_1,172_ = 0.51, *P* = 0.478, estimate: −0.007±0.010 SE) or carotenoid and control fed animals (*F*_1,171_ = 0.39, *P* = 0.533, estimate: 0.007±0.001 SE), and the interaction between carotenoid and corticosterone treatment was not significant (*F*_1,160_ = 0.51, *P* = 0.476). There were no significant differences between populations and none of the interactions were significant (*P*>0.05).

## Discussion

We have investigated the effects of carotenoid availability and blood corticosterone levels on the sexually selected ventral colouration of the common lizard. We first determined the chemical basis of the lizard's bright yellow-orange belly colour. The results indicate that carotenoids are responsible for the colouration. Melanins and pteridins do not appear to contribute to the yellow-orange colour, since no yellow-orange pigments remained in the skin after acetone extraction [61]. The carotenoid-content of the skin was negatively correlated with the hue of the lizard's skin colouration, explaining around 47% of the observed variation. This indicates that measuring skin colouration enables its carotenoid-content to be assessed.

Carotenoid supplementation did not induce any change in colour, although it did lead to an increase in blood carotenoid concentrations. Since the power to find an effect on colouration of similar size (*d*) as that observed in Tschirren *et al.* [4] (*d* = 1.073; 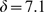) or in Fitze *et al.* [63] (*d* = 0.841; 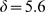) was 100%, our results imply that carotenoid feeding does not affect carotenoid-based colouration in this species, in contrast to studies on birds and fish [4], [12], [18], [64]. This finding is in line with the existence of colour morphs [8] and together with an earlier study, that estimated the heritability of the common lizard's colouration at 0.5 [48], it suggests that the three types of colour morphs described in the common lizard (white, yellow, and orange) may be the result of differential carotenoid-incorporation, potentially explaining how colouration may reveal an individual's life-history strategy [7], [65].

It has been shown that colour signals are usually genetically and environmentally determined [1]–[5]. Environmental determination of the common lizard's colouration has already been proposed [23] and our results show that ventral colouration varies with environmental conditions (i.e.: stress response). Chronically increased blood corticosterone levels negatively affected the hue of the ventral colour in both male and female lizards (shifting towards a redder colouration). Similarly, increased blood corticosterone levels were associated with redder colouration in non-manipulated lizards from natural populations (Figure 3). This indicates that increases in corticosterone, also found during PSR, are not directly responsible for the colour fading observed during stressful situations in the common lizard [22], [23]. The effect of corticosterone on colouration may have been the result of enhanced appetite, potentially leading to increased carotenoid transport and hence, to an increase in colouration. However, the observed colour change in corticosterone-treated individuals was not explained by carotenoid-availability, food consumption or a change in body condition, indicating that the effect of corticosterone was not due to differential food intake. Corticosterone may also provoke a reallocation of carotenoids (e.g. from the liver to the blood), since pigmentation is often under hormonal control (such as androgens or oestrogen) [5]. Such reallocation may occur through the hormones' effects on carotenoid transporting proteins [66] or through direct reallocation of carotenoids from storage sites to the blood stream [67], both resulting in changes of the blood carotenoid concentrations. Since increased concentrations of blood carotenoids did not affect colouration, it is unlikely that the effect of corticosterone on colouration was mediated by carotenoids. Hence, the mechanism by which corticosterone affects carotenoid-based colouration of common lizards remains unknown and investigating these mechanisms should be addressed in future studies.

Whatever the precise physiological mechanism, the results presented here are in line with the ELHS theory, suggesting that chronically enhanced corticosterone levels may activate the ELHS, and given that food was abundant this may have led to positive rather than negative effects (Figure 1 (v); see also [41]). Alternatively, chronic increases in blood corticosterone levels may have changed an animal's life history strategy [35] from long-term to short-term investment. As a consequence, lizards may invest mainly in short-term survival, as shown previously [30], [42]–[44], and in sexual attractiveness by becoming redder. Indeed, in experiments where adult males had the opportunity to copulate with three different females, redder males (smaller hue values) copulated with more females (ANOVA: *F*_2,38_ = 3.535, *P* = 0.039; not copulating: 69.592°±1.182 SE, once copulating: 67.965°±1.841 SE, twice copulating: 58.059°±2.817 SE; for experimental design see [52]), suggesting that lizards may have made a kind of ‘terminal investment’ [68]. These two scenarios are not incompatible since the ELHS might be the physiological mechanism by which the strategy “terminal investment” is activated. However, future studies will be necessary to reveal whether increased corticosterone levels truly have positive effects under circumstances of food restriction.

Our study clearly shows that the ventral colouration of common lizards is partly determined environmentally and it suggests that belly colouration may be a condition-dependent trait. Condition-dependency is in agreement with studies on the colouration of other lizards [46] and it is in line with studies on birds and fish, where carotenoid-based colouration is a condition-dependent trait that honestly reflects an individual's quality [1], [4], [18], [69]. However, our study also suggests that unlike in birds and fish, the condition-dependency may not be due to carotenoid availability. Since carotenoid availability did not affect colouration, the common lizard's belly colour may be a composite trait, with genetic determination of the carotenoid-based element and condition-dependent determination of yet unidentified components, like cell structure, melanophore content or iridophore content [70]. Consequently, females who select redder males are selecting males with increased stress responses but not males with higher carotenoid-availability.
